# *The Cornbelt's Last Open Pollinated Corn*: Agricultural extension and the origins of the hybrid corn seed industry

**DOI:** 10.1002/ppp3.10414

**Published:** 2023-08-14

**Authors:** Helen Anne Curry

**Affiliations:** 1 School of History and Sociology, Georgia Institute of Technology, Atlanta, Georgia, USA; 2 Department of History and Philosophy of Science, University of Cambridge, Cambridge, UK

**Keywords:** agricultural extension, demonstration, hybrid corn, open-pollinated varieties, plant breeding, seed industry

## Abstract

Agricultural extension is recognized as an important pathway for generating changes in individual farmers' practices and therefore broader patterns of production. In the United States, historical research has implicated extension work in transformations that privileged White farmers and wealthier operations over other producers and that fostered the industrialization and consolidation of farms. This article examines the work of one early 20th-century extension agent and the demonstrations he used to teach farmers how to choose and keep corn seeds and to identify the best performing corn varieties for a particular location. This history can inform contemporary efforts to develop more socially and ecologically aware approaches to agricultural research, extension, and production by emphasizing the need for measures of success that align with community-level objectives and for larger institutional structures that support and sustain such goals.

**Summary:**

- The article examines the histories of agricultural extension and crop development in the early 20th-century United States. It discusses the role of farm demonstrations, including the participation of farmer-breeders, in the development of spread of higher yielding corn varieties in the Midwestern states in the 1910s and 1920s. It highlights the emphasis placed on finding locally or regionally appropriate varieties in some early corn extension activities and dwells on the irony that these locally specific endeavors played a role in the development of universalized solutions.
- The article examines and contextualizes an unusual archival document as an entry point into these histories: *The Cornbelt's Last Open Pollinated Corn*, a two-volume work prepared by Martin Luther Mosher (1882–1982). Mosher was the first county agricultural extension agent in the state of Iowa and worked in extension until his retirement in 1950.
- The article makes three main observations: (1) *The Cornbelt's Last Open Pollinated Corn* is best read as an agricultural demonstration; (2) *The Cornbelt's Last Open Pollinated Corn* is Mosher's attempt to grapple with the material legacies of his extension work in relation to the different agricultural life he idealized; and (3) Mosher's work exemplifies the complex relationships and expectations seen among breeders, seed companies, extension agents, and farmers in the early 20th-century United States.
- The article concludes that Mosher's work with open-pollinated corn varieties offers insight into the importance of agricultural extension as a means of crop development and highlights the contingent nature of agricultural industrialization.

## 1 Introduction

**Figure 1:**
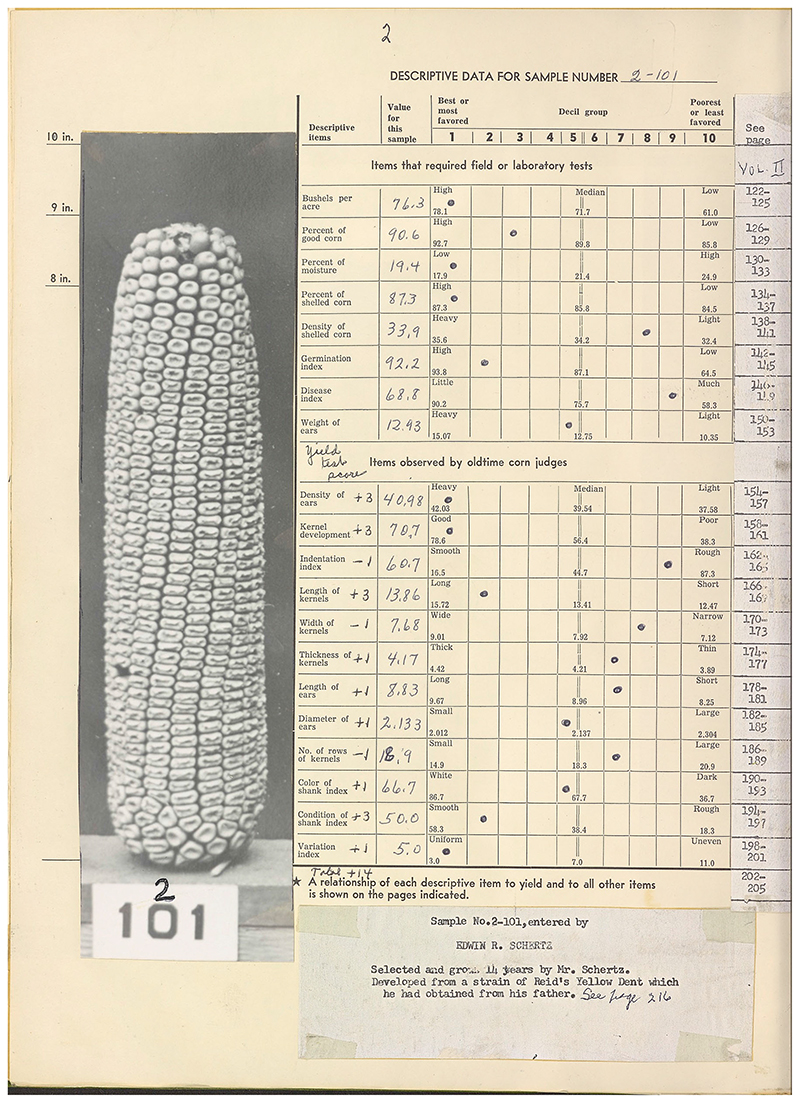
A farmer's variety of open-pollinated corn reproduced in Martin Mosher's volumes to reflect its actual size, with accompanying data for that variety from the Woodford County corn yield test. From Mosher, *The Cornbelt's Last Open Pollinated Corn*, vol. 1, p. 2. https://archive.org/embed/cat74417289001.

In 1974, at age 92, Iowa resident Martin Luther Mosher completed his two-volume *The Cornbelt's Last Open Pollinated Corn* and deposited a copy with the US National Agricultural Library.^1^ These hand-assembled volumes reproduced life-size photographs and descriptions of corn types used for seed by White settler farmers of the Midwestern United States in the 1910s. (Figure 1). Mosher, who had been the first county agricultural extension agent in Iowa (appointed in 1912) and subsequently worked in extension in Illinois (from 1916 to 1950), emphasized the value of the historic records gathered in his books. He believed they uniquely illustrated the typical maize varieties grown by settler farmers prior to the introduction of hybrid corn and therefore gave access to biological and social forms long since abandoned (Mosher, 1974). What motivated the elderly Mosher to spend, by his own accounting, “four to six hours per day here at my desk working on these corn books,” over a period of 3 years? (Mosher, 1974, vol. 2, p. 225). Nostalgia may have been one motivator, and a life-long commitment to thorough record keeping was clearly a second. Yet these are not fully satisfying explanations for a work as unusual as *The Cornbelt's Last Open Pollinated Corn*.

Mosher's life spanned a century of profound transformation in farming and rural communities in the United States. He experienced the booming farm economy of the first two decades of the 20th century, the crushing depression that followed World War I, and the adoption of government support as a permanent fixture of US agricultural policy in the New Deal of the 1930s. He saw farms become more mechanized, more capitalized, and more dependent on off-farm inputs in subsequent decades. He witnessed the rise of large-scale agribusiness as the dominant form of farming enterprise and the steady outmigration of Americans from the countryside to cities (Danbom, 2017; Fitzgerald, 2003). As an agricultural extension agent for nearly five of these turbulent decades, Mosher participated in programs that aimed to foster rural development through direct engagement with farm families (Sullivan, 2021; see also Schwieder, 1993; Pellack & Karlen, 2017; Vail, 2018). In this paper, I explore the relationship between Mosher's extension work and the dramatic transitions he lived through. Reading archival documents and records from his early career alongside his retrospective accounts, I reveal a contradiction in the goals Mosher espoused and the outcomes he helped produce. Building on this observation, I suggest that his story offers broader lessons for thinking about the work of extension.

Examining Mosher's early career also explains his commitment to The Cornbelt's Last Pollinated Corn. The text underscores the value he placed on demonstration as an instrument of farmer understanding and agricultural transformation. He evidently remained committed to demonstration—an approach to agricultural extension in which agents persuade farmers to adopt new ideas and methods by offering them direct experience—decades later. I argue that *The Cornbelt's Last Open Pollinated Corn* is best read as a demonstration: It educates readers in Mosher's version of the history and legacy of extension in the Corn Belt by leading them step by step—and corn ear by corn ear—through an influential crop research and extension program. Mosher's exposition does not push readers to lament the loss of open-pollinated corn, as one might expect from the title, but instead to wonder about the abandonment of public agricultural services tailored to local social and ecological needs.

Studying Mosher's volumes in turn highlights an irony that perhaps troubled him enough to serve as additional motivation for the book's creation. The very same programs of locally specific crop development that he championed, and which are the central object of *The Cornbelt's Last Open Pollinated Corn*, facilitated the spread of monocultures that eventually rendered such programs obsolete (Fitzgerald, 1990). In other writings, Mosher mourned the disappearance of rural opportunities and disintegration of communities as farms scaled up and consolidated in the middle decades of the 20th century (e.g., Mosher, 1962b). I suggest that *The Cornbelt's Last Open Pollinated Corn* may also be read as his attempt to grapple with the material legacies of his extension work in relation to the agricultural life he idealized. Mosher possessed an exaggerated understanding of his influence within events driven largely by commercial interests, state authority, and changing technical possibilities. However, this heightened sense of his own significance spotlights the potential tensions between individual aspirations and institutional possibilities in the realm of agricultural extension.

Historians have addressed the pivotal role of professionalized plant breeding in the reorganization of crop research and agricultural production across diverse political regimes in the 20th century (e.g., Bonneuil, 1999; Charnley, 2011; Harwood, 2012; Saraiva, 2016; see also the literature review in Berry, 2021). Scholars focused on the United States have persuasively shown how commercial production of hybrid corn seed in the Midwestern states contributed to the growth of the private seed industry and diminished both public scientists' role in crop development and farmers' control over seeds (Fitzgerald, 1986, 1990, 1993; Kloppenburg, 2004). Historical studies have also implicated agricultural extension as an instrument of industrial agricultural transformation and entrenchment (Guthman & Zurawski, 2020), for example, by showing how in the United States extension agents consistently oriented their work towards the needs of large farm operations rather than small-scale producers (Henke, 2008).^2^ The industrialization of agriculture in the US Corn Belt and beyond resulted from many factors (Anderson, 2009; Danbom, 2017). Extension many have been more marginal to this shift than state policies favoring commodity producers or the availability of and advocacy for agrochemicals. Nonetheless, it was and is still seen as having been a powerful tool of rural transformation (Danbom, 1979; Fitzgerald, 2003; Rosenberg, 2016).

I situate *The Cornbelt's Last Open Pollinated Corn*—a historical account of crop breeding and extension prepared by a breeder and extension agent—within these established historical frames. My aim is not only, or even chiefly, to understand why Mosher prepared his account. I instead want to revisit the relationships among breeders, seed companies, extension agents, and farmers at a time when the boundaries between these roles were fluid, to examine whose knowledge and skills were considered valuable to the project of agricultural advancement in the Corn Belt.^3^ My analysis highlights in particular the ground-level experiences of this work, from the perspective of an extension agent who was more or less improvising interventions in these early years of institutionalized extension. I suggest that this close study of an important series of farm demonstrations highlights the contingent development of agricultural extension, “improved” corn, and private seed companies—and through these the contingent, rather than inevitable, industrialization of the US Corn Belt.

## 2 Farm Extension For Agricultural Improvement

In 1906, Martin Luther Mosher, recently graduated from the Iowa State Agricultural College in Ames, Iowa, joined the college's newly organized department of agricultural extension. The department's existence testified to a growing belief that moving knowledge developed at the college and experiment station to farmers' fields required dedicated efforts (Rogers, 1988). Growing up on an Iowa farm, Mosher had attended farmers' association meetings and institutes with his father where he had listened to discussions of the day-to-day challenges of raising crops and livestock (Mosher, 1935, September 15). As a college graduate and professional agronomist, he now found himself on the other side of the podium, delivering information about best practices at meetings rather than receiving it. The swap proved genial. Although as a young man he had intended to start his own farm, Mosher continued in agricultural extension for the rest of his career. His early experiences in the field were critical in establishing his understanding of the economic and social value of extension work, especially demonstrations where farmers could see firsthand the effects of adopting different practices. Here, I discuss Mosher's involvement in several well-documented extension efforts in Iowa early in his career. These programs illustrate the initial institutionalization and professionalization of extension in the United States, the methods Mosher and others considered effective for gaining information from and delivering information to farmers, and the early entrenchment of a narrative celebrating extension's role in the “modernization” of farms and farmers of the Midwestern United States.^4^

At the department of agricultural extension, Mosher worked under Perry Greeley Holden, who had been hired in 1902 as the college's vice dean of agriculture. Prior to arriving in Iowa, Holden had experimented in corn breeding, led field research for a sugar refiner, and run farmer education programs for a seed company (Sizer & Silag, 1981). At the Iowa State Agricultural College, Holden's responsibilities included educational outreach to farms—that is, extension— at a time when this had yet to become a systematized component of state and national agricultural programs (Schwieder, 1993; Scott, 1970). (Greater systematization would come with the Smith–Lever Act of 1914, the federal law which established the state-national agricultural extension service.) Holden gave lectures on seed care and cultivation to farmers, set up a system of demonstration plots where they could observe effects of different practices on crop quality and yield, and held corn shows where neighboring growers could compare their products (Pellack & Karlen, 2017; Sizer & Silag, 1981). Holden's commitment to reaching the state's far-flung rural communities on their own turf, rather than at the college or experiment station, inspired the “Seed Corn Special” (also known as the “Corn Gospel Special”) (Vail, 2018). Launched in 1904, this train service delivered lectures and exhibits on corn cultivation to audiences across the state over the next 3 years, eventually reaching 97 of Iowa's 99 counties and an estimated 145,000 people (Sizer & Silag, 1981, p. 69; Vail, 2018).

**Figure 2:**
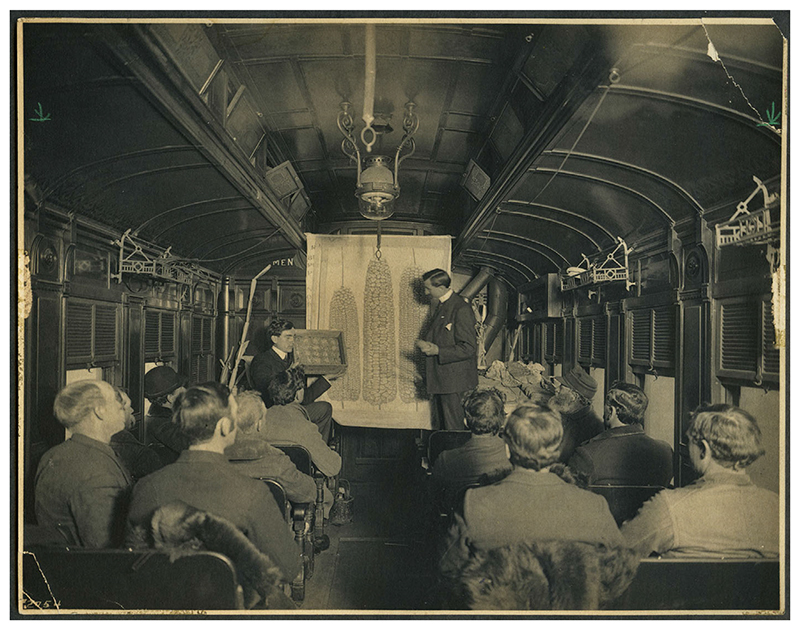
All aboard the Seed Corn Special! While his colleague J. W. Jones lectures, Martin Luther Mosher (seated) holds aloft a corn seed germination testing box. Perry Holden recommended that farmers use a testing box to determine the quality of seeds prior to planting, 1905. https://n2t.net/ark:/87292/w96m0c. By permission of Iowa State University Library Special Collections and University Archives.

It is unlikely that the Seed Corn Special affected Iowa corn harvests as directly as its champions maintained. Farmers' presence for a lecture on seed selection was hardly evidence of their changed behavior in the fields. Instead, the vigorous response of farmers in showing up to listen helped Holden make his case to the Iowa General Assembly that it ought to establish a department of agricultural extension at Iowa State and install him as its first director in 1906 (Sizer & Silag, 1981, p. 70). The Seed Corn Train also affected Mosher, who participated in the venture as a lecturer during his senior year at Iowa State (Figure 2). That year, over a period of 10 weeks, the Seed Corn Special stopped at 10 to 15 towns daily for a total of about 600 towns. At each stop, expectant farmers were invited on board to listen to lectures. Mosher (1935, October 6) considered this experience his “real entrance into extension work.” After a short post-graduation teaching stint, Mosher left Ames, returning when Holden beckoned him in 1906 to join the now institutionalized program of Iowa-wide extension.

On Mosher's return, Holden charged him with running the department's “county farm demonstrations.” These demonstrations aimed to teach farmers the outcomes of different varieties, cultivation methods, or other agricultural practices through direct observation and firsthand experience.^5^ When the Iowa legislature had established the state's department of extension in 1906, it permitted the board of supervisors of any Iowa county to appropriate $300 to fund demonstrations conducted by the new department on that county's public farm. It was now Mosher's task to follow up wherever county boards were interested. As he described, his work focused on corn and typically involved four demonstrations: the farmers' variety test, the introduced variety test, the individual ear test, and the thickness of planting test (Mosher, 1936, January 12). Each was imagined to impart a lesson or two to farmers, but often provided useful local data to the demonstrator and to the state extension service as well (Mosher, 1962a, p. 16).

**Figure 3:**
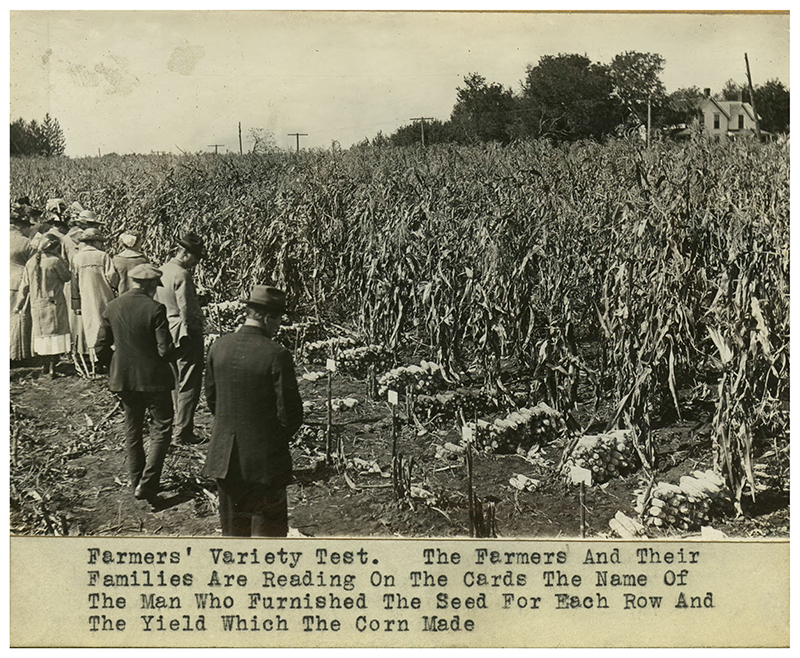
The farmers' variety test demonstration allowed farmers and their families to see differences in corn health and yield among varieties maintained and grown in their local area. Farmers' variety test, Iowa, 1918. https://n2t.net/ark:/87292/w9f34w. By permission of Iowa State University Library Special Collections and University Archives.

The farmers' variety test was, in Mosher's estimation, the most important of the demonstrations. Its main purpose was to illustrate for farmers the variable quality of corn seeds planted across the county, with visual displays and quantitative comparisons of final yields driving home the lesson that good harvests were not entirely about the skills of farmers, the conditions of their farms, or the local weather conditions, although these obviously mattered. They also depended on the nature of the seeds planted (Figure 3). It was essential to the relevance of the demonstration that it rely on the actual seeds used by farmers. Mosher therefore had “interested men” traverse the county at early planting time “to get a quart of seed corn from the planter-box whenever a man was found planting,” a collection that typically resulted in 60 to 100 samples. These were then planted on the county farm in small plots, where they would be visible to farmers who attended extension events organized at the farm in the autumn. The plots were harvested so that the yield obtained from each individual farmer's seeds could be tabulated and compared (Mosher, 1936, January 12; see also Mosher, 1962a, chapter 3). Through the farmers' variety test, repeated from county to county, Iowa farmers were meant to learn that they sometimes brought in less corn “only because they were planting less productive seed than some of their neighbors” (Mosher, 1962a, p. 30). As a result, it encouraged some farmers to seek further instruction, for example, on how to select healthy seeds from an ear, test them for quality, and plant them most effectively.

The demonstrations also gradually reshaped the advice on selecting corn seed that extension agents gave to growers. The data generated in the repetition of the farmers' variety test revealed to Mosher and his colleagues the extent to which factors other than good germination and effective planting influenced yield. As Mosher (1915, pp. 1–4) summarized, “In every community where corn is a main crop, some man is using seed which is capable of producing several bushels more corn per acre of as good or better quality [of corn] than the average seed used in the community.” In other words, some farmers' varieties were simply more productive. The data aggregated from repetition of the other demonstrations was equally revealing. The imported variety test, which compared seeds from large seed companies and “the supposedly best seed corn growers in various parts of the state” to those of farmers in the county where the test was conducted, showed that “local” seeds (i.e., those from the county) typically outperformed “imported” seeds. The individual ear test, which compared the performance of seeds obtained from several different ears of the same farmer's corn, confirmed that some ears were “inherently” higher yielding than others. These conclusions suggested that extension agents' advice to farmers on seed quality would be more effective if, in addition to addressing seed care and testing, it included the identification and circulation of the local seeds that yield and ear tests revealed to be more productive (Mosher, 1915; see also Mosher, 1962a).

Organizing a demonstration that could accomplish all these tasks was difficult, especially when one's responsibilities included farmers across the state. However, a change in Mosher's assignment soon created new possibilities. By 1910, several US states had instituted county-level extension services managed by designated county agricultural extension agents (Rogers, 1988, pp. 496–497). Mosher became enthusiastic about this approach to organizing extension. He recommended it to his superiors, simultaneously putting himself forward as a candidate for county agent. In 1912, both prospects materialized: Mosher became the Clinton County agricultural advisor, the first county agricultural extension agent in the state of Iowa (Mosher, 1962b; Schwieder, 1993).

Mosher assumed his new role with a plan for integrating corn demonstrations into a system of seed production. The number one action on his 1912 list of “Some of the Things That a County Expert Would Do” was identifying *and distributing* better corn varieties (Mosher, 1912b). This process would start with the typical farmers' variety yield test, featuring the varieties planted by Clinton County settler farmers who relied on their own seeds. It would be followed up with a second and third year of tests in which the pool of compared varieties was gradually narrowed to the best performing ones as measured by yield. After 3 years of assessment, the most consistent high yielder would be confirmed, and arrangements made for seed from that variety to be multiplied and made available to local farmers. Those farmers could then plant and maintain the variety on their own land moving forward.

**Figure 4:**
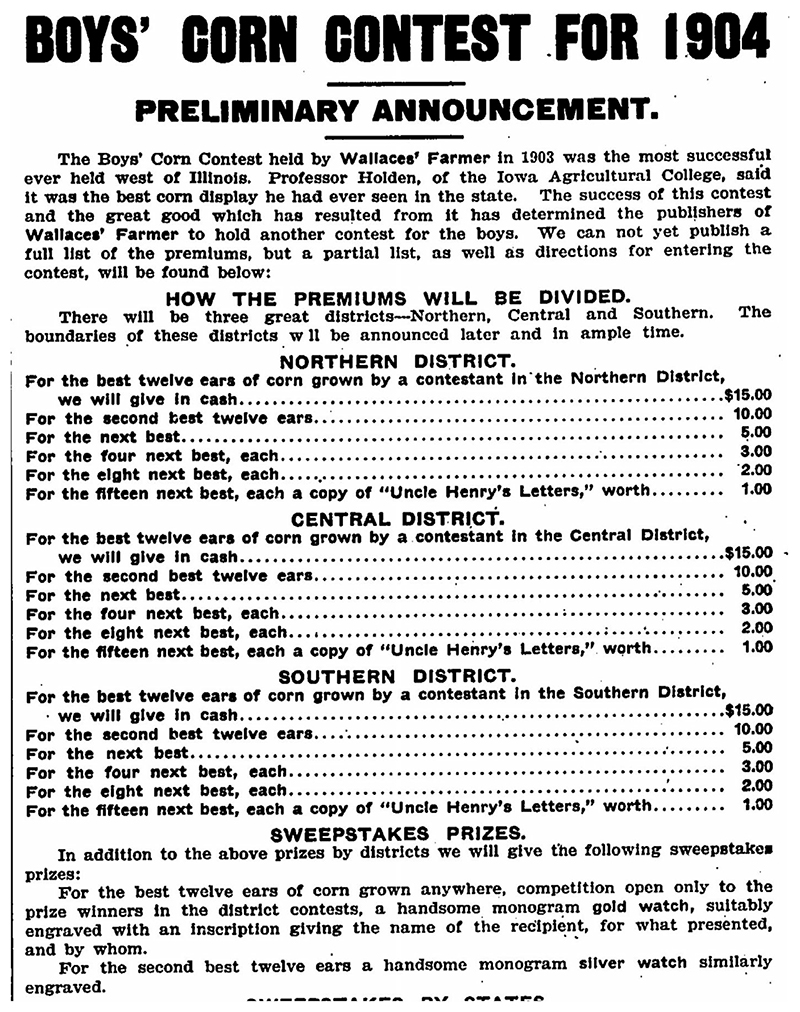
The weekly newspaper *Wallace's Farmer* bolstered extension work by advocating that farmers pay attention to the origin and quality of the corn seed they planted. In the early 1900s, it orchestrated corn-raising competitions for boys, such as the one advertised here. *Wallace's Farmer*, January 8, 1904, p. 41. Illinois Digital Newspaper Collections, https://idnc.library.illinois.edu.

The Clinton County corn yield test proceeded along these lines starting in 1913 (Mosher, 1962a, chapter 7). By 1915, Mosher had ranked the top 5 performers. Despite dominating in the quantifiable attributes of yield and early ripening, the Leaming corn kept by farmer C. H. Joehnk was deemed too difficult to handle without mechanical pickers. The next highest yielding variety was disqualified on the grounds of having only been tested two of the three years. The next three varieties had nearly identical yields, but one was slower to ripen and therefore relegated to fifth. By these machinations, Mosher declared the varieties grown by C. W. Greve and A. H. Studeman as most suitable for general use across the county. These varieties' comparable performance had an obvious explanation: Not only were they both early-maturing strains of Reid's Yellow Dent corn, a popular and reliable variety in wide circulation since the 1890s, but they had also both originated in seed samples distributed around 1903 by the influential Iowa farm weekly *Wallace's Farmer* as part of a corngrowing competition for boys (Mosher, 1962a, pp. 71–74) (Figure 4). The samples had then been maintained by Greve and Studeman on their farms and potentially adapted modestly to local conditions in the intervening years. Ultimately, it was the Studeman variety that served as the foundation of a multiplication and distribution program led by the county farmers' association (Mosher, 1915).

Mosher's efforts transformed the composition of local corn fields. By the early 1920s, an estimated two-thirds or more of Clinton County farmers were raising the “Studeman strain” of Reid's Yellow Dent corn (Anonymous, 1922; see also Mosher, 1962a, p. 74). Although in hindsight Mosher's Clinton County test looks like a clear case of institutionalized extension services targeting only the narrow metric of yield with the result of further entrenching standardized monocultures and industrial aspirations, looking forward from Mosher's position in the 1910s, a different possibility comes into view. He understood himself to have improvised a new mechanism for identifying and distributing the best-adapted local corn seed throughout a locality and took satisfaction in farmers' evident appreciation of the better quality seed now available for them to continue growing and adapting on their own farms.

In the years after Mosher's corn test, Clinton County farmers either realized the value of the Studeman strain in the form of increased harvests, and stuck with it for that reason, or were simply persuaded by the elaborate demonstration that Studeman's seeds were better than whatever they had grown before.^6^ In either case, Mosher's activities appeared to have served the extension agenda of changing farmers' practices to align with research-based recommendations. Whether they advanced other objectives that Mosher (1912a, pp. 16–17) embraced for county extension work is more difficult to assess. He had emphasized in 1912, at the start of the project, that “[t]he real reason for all of the work … is the betterment of community life and home life.” As he explained:

The increasing of crop and animal production and the increase of money profits by these means will be of no avail if such money is not used to make the homes more pleasant and attractive, to improve the schools, the rural churches, the roads, social life in the country and all other things which have a part in making the home and community life more pleasant.

Many early 20th-century boosters for agricultural extension such as Cornell University professor Liberty Hyde Bailey shared Mosher's vision of extension as a vehicle for more than just material or economic gain (Peters, 2006). The broader Country Life movement whose goals they espoused sought to make agriculture more efficient and therefore more sustaining of urban industrialization (Danbom, 1979) but was equally concerned with rooting a white, Christian rural population increasingly perceived to be fleeing the limitations of life in the countryside (Danbom, 2017, pp. 156–57; see also Schwieder, 1993). How did Mosher's demonstrations contribute to these aspirations for rural communities? His next county-wide test—and his own gloss on it at 50 years' remove—offers insights into the complex legacies of the corn-yield test he championed.

## 3 Seed Quality Versus Yield Quantity

The Clinton County yield test, itself inspired by the earlier yield demonstrations across Iowa counties, generated influential follow-ons. In Iowa, it is said to have encouraged the institution of a state-wide corn yield test. First held in 1920 thanks largely to the efforts of Henry A. Wallace of *Wallace's Farmer* (later the Secretary of Agriculture and eventually Vice President of the United States) and H. D. Hughes of the Iowa State Experiment Station, the Iowa corn yield test brought together farmers' seeds from across the state. These were then grown under comparable conditions, with prizes awarded based on performance (Pellack & Karlen, 2017, pp. 1987–1988; see also Robinson & Knott, 1963). Wallace—who had lived in the same house with Mosher as a college student and helped him conduct at least one of his county demonstrations in Iowa—later credited Mosher and Holden with creating the “mental climate” conducive to the success of the later Iowa corn yield tests (see introduction by Wallace in Mosher, 1962a, p. 5). Meanwhile, in neighboring Illinois, the corn growers of Woodford County benefited directly from knowledge and skills gleaned in the Clinton County demonstrations when Mosher arrived to take a position as county farm adviser in January 1916. The Woodford County corn yield test of 1919–1922, like its predecessor in Clinton, sought to identify and distribute the “superior strains” of corn being grown in the county (Mosher, 1962a, chapter 8). These and other corn yield tests have been identified as important in the mid-20th-century transformation of the US Corn Belt (Pellack & Karlen, 2017; Robinson & Knott, 1963). As I describe here, they directly contributed to the development and adoption of hybrid corn seeds, which would eventually be produced and sold exclusively by seed companies. Within a couple of decades, hybrid seeds would eliminate the need for farmer education in seed quality and care (Fitzgerald, 1993)—education which Mosher and his colleagues had considered essential to the improvement of corn, and rural life, in the Corn Belt.

Although Mosher arrived in 1916 to Woodford County, where leading members of the community sought a farmers' variety test of the kind carried out in Clinton County, his implementation of such a test was delayed by the world war. It was not until January 1919 that 118 men from the county each arrived at a local meeting with 100 ears of the corn he intended to use as seed for the next season, finally launching the effort. These settler farmers were, as Mosher later characterized them, “ordinary good farmers … growing corn for feed on their own farms or for sale as grain.” All but a few had been growing their variety in the county for 5 or more years. They were not men who “show[ed] corn at the local, county, state or national shows” (Mosher, 1962a, p. 77; Mosher, 1974, vol. 1, interior cover), venues where since the 1890s farmers had been rewarded for producing ears that conformed to visual standards for a “good” ear or the ideal type of a particular variety (Fitzgerald, 1993). In other words, the seeds contributed by the farmers in the Woodford County test were local, typical, and intended for productivity, not “imported,” purchased from a seed seller, or bred for display.

After assessing the ears together, Mosher and the farmers set aside the 20 ears in the poorest condition and selected another 10 “typical” ears for display. The rest provided the seed samples that would be planted in the yield test: The farmers selected three rows of kernels from each of the 70 ears (with the rest of the ear returned to the farmer). These were then further tested for germination, ear sample by ear sample, and the ones deemed healthiest were advanced to the trial. By growing the samples in four different experimental plots, sowing by hand and always using the same number of seeds per hill, Mosher created experimental conditions to facilitate comparisons among the different farmers' varieties, just as he had in Clinton County (Mosher, 1962a, pp. 77–78).

After following this procedure over three years, losing only three participants who had either left farming or the county in that time, the Woodford County corn yield test seemed once again to bear out Mosher's view that there was, in every place, one farmer whose corn was just plain better than everyone else's. In Woodford County, that farmer was George Krug. A “quiet, retiring farmer who kept pretty much to himself,” Krug had at some point mixed a Nebraskan strain of Reid's Yellow Dent corn with a variety called Iowa Gold Mine (Wallace & Brown, 1956, p. 74). He selected seeds from that mix for the next 14 years. When choosing ears to use for seeds each season, he followed many of the instructions that Holden, Mosher, and other extension agents so zealously imparted, such as only taking ears from plants that had been healthy in the field and looking for the plumpest kernels of those ears.

**Figure 5:**
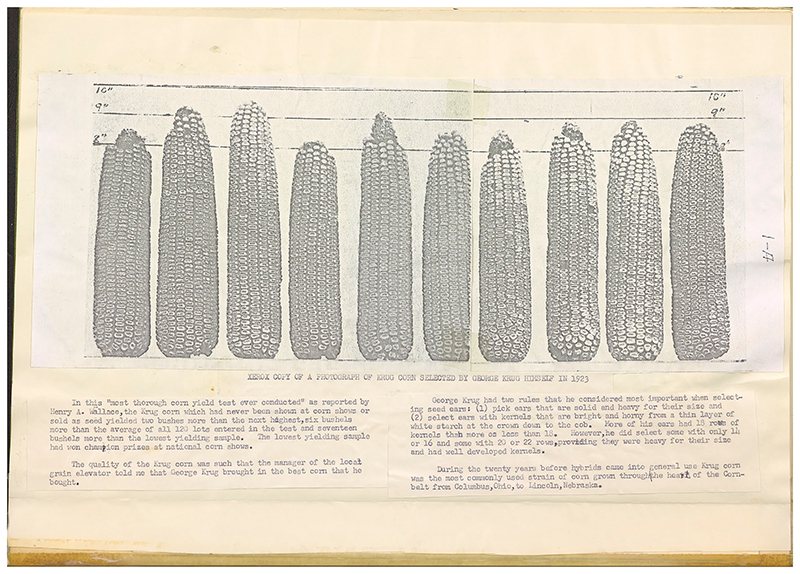
“Xerox copy of a photograph of Krug Corn selected by George Krug himself in 1923.” This record of Krug's corn, preserved by Mosher, illustrates some of its variability, for example in the size of the ear. Mosher's notes below relate that Krug's rules for selecting seeds prioritized the heft of the ear and the visual appearance of the kernels once removed from the cob. From Mosher, *The Cornbelt's Last Open Pollinated Corn*, vol. 1, p. 1-A. https://archive.org/embed/cat74417289001.

Mosher later recalled Krug's corn variety as visually unimpressive, its ears and seeds variable and uneven in comparison to other farmers' more uniform offerings (Wallace & Brown, 1956, p. 74). (Figure 5). However, its superlative field performance in terms of yield was undeniable. In the 12 field trials (four plots planted in each of the three years), Krug's variety landed in the top 10% eight times. It produced a 3-year average harvest that amounted to 1.8 bushels more per acre than the next ranking competitor and 6.6 bushels more than the overall average harvest. In 1922, when it was included in a further comparative test, this time featuring the top 12 Woodford County farmers' varieties and two popular commercial varieties produced in the region, it again came out on top. Neither the “widely advertised and very good ‘disease free’ corn” sold on the market nor the strain that was the “consistent winner” of the Illinois Utility Corn Show could compete with Krug's variable but vigorous variety when planted in Woodford County (Mosher, 1962a, pp. 78–81).

In 1919, as the farmers' variety yield test was getting underway, the Woodford County Agricultural Association—later the Woodford County Seed Company—had established a system for producing and selling whatever variety would be identified as highest yielding. At the test's conclusion, the association negotiated a contract with Krug to buy seed that he personally selected from his crop. This seed, perhaps a few hundred bushels, was then “put out to good corn growers under contract” and it was these contracted farmers who created the seed corn that the association sold on to other farmers. Krug reportedly got a good price for his initial seeds. He also received a small royalty on the proceeds of the commercial seeds garnered by the association (Mosher, 1962a, pp. 82–83). Did farmers reap any benefits? Although growing a higher-yielding variety and thereby bringing in more bushels of corn would have seemed a boon to those farmers producing corn as a cash crop, the Woodford County test concluded in the initial years of what would prove to be a long-lasting agricultural depression (Danbom, 2017). At the scale of counties, states, and the nation, producing more grain only drove already low prices lower.

In the absence of government aid, individual growers nonetheless had little choice but to pursue productivity, hoping that a bigger harvest would offset falling prices. Publicity around the Woodford yield test and its results led to high demand for the victorious variety, and not just among Woodford County growers. Private seed companies also bought supplies from the Woodford association to use as foundation stock for distribution beyond the local area. Growers of Krug corn in other counties soon came out on top in their local variety tests and shows, further driving demand. Krug's variety and derivations of it rapidly made their way into fields across the central Corn Belt. According to Mosher, who clearly had a stake in reporting such an outcome, Krug corn was, by the early 1930s, “the most generally grown of any single strain of open pollinated corn in an area from 50 to 100 miles wide across the south central part of the Corn Belt from Ohio to Nebraska” (Mosher, 1962a, p. 87). On the matter of how this result related to the variety test's ostensible purpose of showing the value of farmers' local varieties over seeds “imported” from other areas, he remained understandably but unhelpfully silent. His yield test had ramified in unexpected ways.

Even as Midwestern farmers adopted Krug's champion strain, the attention of breeders and growers was turning away from farmers' varieties. For years, farmers and breeders had observed that crossing two lines that had been inbred over multiple generations often produced vigorous, high yielding, “hybrid” offspring; however, profitably producing hybrid corn seeds had remained a challenge until genetic researchers proposed an effective workaround in the late 1910s. By the 1920s, many people were interested in developing—and profiting—from hybrid seeds (Fitzgerald, 1990, 1993; Kloppenburg, 2004). In comparison to open-pollinated farmers' varieties like the ones maintained by Studeman and Krug, in which individual corn plants genetically intermingled through free cross pollination in the field and farmers selected and maintained their corn's desirable qualities at the population level, hybrid varieties were the product of controlled cross pollination (and thus controlled genetic mixing) of specific inbred lines. Qualities thought desirable in the hybrid variety were maintained in the inbred parent populations. There was therefore no role for the farmer in shaping and maintaining a hybrid variety growing in the field in the way that Krug had crafted and kept his open-pollinated variety. Because hybrids produced highly variable seeds thanks to their heterogeneous parentage, farmers needed to return to the keeper of the original inbred lines for a fresh batch of hybrid seeds each season. When those keepers were professional breeders or private companies, this eliminated even the need for strategies of seed selection and care, including the lessons once imparted on the Seed Corn Special (Fitzgerald, 1993; on histories of hybrid seeds see Curry, 2023).

One especially notable hybrid enthusiast was Henry A. Wallace, who developed the hybrid variety Copper Cross in 1923 (and then contracted for it to be produced and sold) and founded his own hybrid seed company, Pioneer Hi-Bred, a few years later (Sutch, 2008; see also Wallace & Brown, 1956, chapter 13; Brown, 1983). Another was Lester Pfister, a Woodford County farmer who'd spent several years painstakingly selecting a white corn variety that he'd entered into Mosher's county-wide yield test in 1919. Mosher had taken note of Pfister's attention to detail, first hiring him to help weigh and score the corn for the test and then turning over the task to him entirely. In 1922, Pfister started to grow and sell Krug corn, reportedly selecting the seeds each season according to the same rules of thumb that Krug had used to develop the variety. He was soon winning corn yield competitions himself, including the state-wide Iowa corn yield test in 1926. When he began developing hybrid lines for sale a few years later, an enterprise that grew into one of the Corn Belt's leading seed companies, Krug-derived inbreds underpinned his product line (Anonymous, 1970; Mosher, 1962a, pp. 85–89; Smith et al., 2004, p. vii). Pfister was not unique in his approach. Across the Corn Belt, would-be producers of hybrid varieties looked to the winners of farmers' variety yield tests as the starting point for their inbred lines (Robinson & Knott, 1963, p. 84), and Krug corn numbered among the handful of lines that became dominant in those early years (Anderson, 1944).

The Iowa corn yield test, which already served as a key site for advertising the best of farmers' and seed sellers' open-pollinated varieties, soon became a vehicle for testing and showcasing the best hybrid varieties. These were less often developed by farmers who used the varieties on their own farms than by individuals aspiring to profit from seed selling. The first hybrid to win the Iowa corn yield test was Wallace's Copper Cross, in 1924 (Pellack & Karlen, 2017, p. 1988), and a separate category for hybrids was introduced in 1926. Hybrid entries skyrocketed, from 1 in 1923 to 10 in 1925 to 206 in 1927 and over 1200 a decade later (Mosher, 1962a, p. 94; Robinson & Knott, 1963, p. 77). With hybrids always outperforming open-pollinated varieties overall in terms of yield, the Iowa corn yield test ultimately served as a demonstration to farmers—a category increasingly dominated by those with mechanized, capitalized, commodity-focused operations able to survive the agricultural depression through economies of scale—of why they ought to annually pay a seed company for a supply of hybrid corn seeds. By 1938, hybrid entries out-numbered open-pollinated ones by more than two-to-one. With hybrids rapidly dominating the corn fields of Iowa and the entire Midwest, the open-pollinated category was eventually abandoned (Robinson & Knott, 1963, pp. 84–85).

In most retrospective accounts, the corn variety tests of the early 1900s, which aimed to demonstrate to farmers why they ought to take care in selecting and keeping their seeds, paid even bigger dividends when they identified the most productive open-pollinated varieties. Those open-pollinated varieties in turn became the promising starting points of inbred lines that would be recombined as hybrid varieties. In other words, the variety tests came to be celebrated for their contributions to eliminating the need or even the possibility of farmers to select and care for seeds. As I have explained, those tests were originally intended to generate the opposite effect—that is, to develop farmers' knowledge of and commitment to good practices in selecting, storing, and planting local seeds. In the end, they not only facilitated the rapid spread across a wide region of a handful of corn varieties believed to be superior, but contributed to the burgeoning hybrid seed business and therefore to circumstances where only specialist seed producers, rather than farmers, would be in a position to develop and maintain those varieties.

## 4 Conclusion: History And Demonstration

When Mosher revisited his records of the Woodford County corn yield test in the 1970s, as an elderly man who'd spent his life working and raising a family in the Corn Belt, he did so with full conviction that this and other yield tests had contributed significantly to the development of hybrid corn and the corn seed industry. In focusing on his own experiences, Mosher's assessment neglected the many economic, technical, and regulatory factors that facilitated the development and adoption of hybrid seeds. Yet this inflated estimation led him to a more complex understanding of the legacies of the corn yield tests and extension work more broadly.

**Figure 6:**
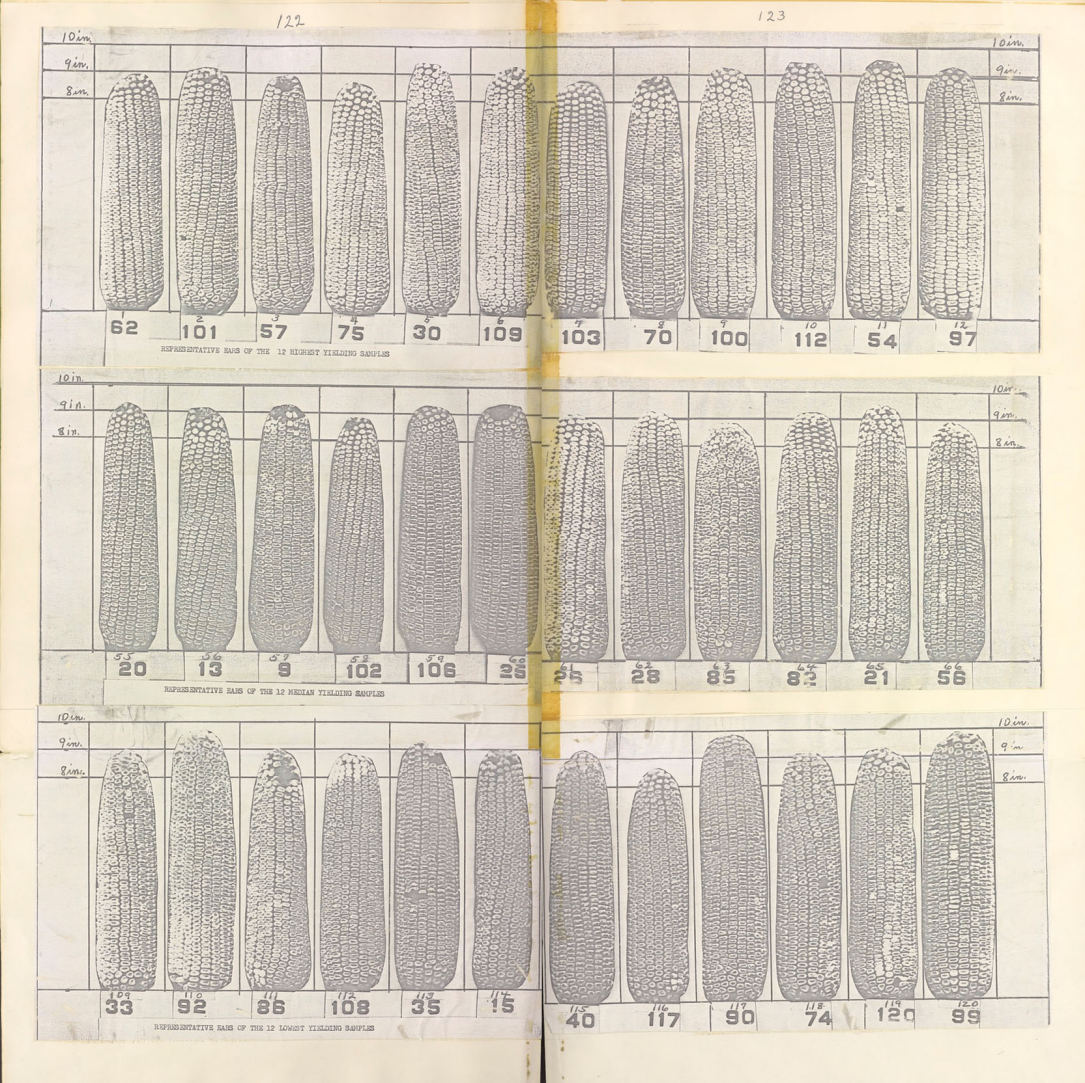
One of many dozens of visual comparisons of maize varieties entered into the Woodford County corn yield test created by Mosher for *The Cornbelt's Last Open Pollinated Corn*. From Mosher, *The Cornbelt's Last Open Pollinated Corn*, vol. 2, pp. 122–123. https://archive.org/details/cat74417289002.

As he prepared *The Cornbelt's Last Open Pollinated Corn*, Mosher returned to each farmers' variety featured in the test, assessing it by the visual standards that had preceded yield tests as well as the measures of yield and weight that he and his colleagues eventually advocated. His aim was not to double-check the results tabulated five decades earlier, but to showcase a world he considered lost. He believed that the photographs he had taken of the 118 varieties “grown by ordinary good farmers” during the Woodford County yield test represented the only extant visual record of what settler farmers had planted prior to hybrid corn (Figure 6). In the intervening years, those varieties had all but disappeared. In 1974, he knew “of only three men in the cornbelt who still grow open pollinated corn” (Mosher, 1974, vol. 1, inner cover; on the history of this transition, see Curry, 2022).

The rapid transition to hybrids had foreclosed several futures. These included the identification of further outstanding farmers' varieties like the Studeman and Krug lines through yield tests, and by extension the use of such varieties as inbred parent lines in hybrid seed production (Mosher, 1974, vol. 2, p. 220; see much earlier discussion of this issue in Anderson, 1944). More important to Mosher was the abandonment of the farm-based practices that he and his colleagues had advocated on the Seed Corn Special and in their early extension work. He judged that the better performance of hybrid corn in the 1930s and later was attributable to more than just their genetic constitution and purported hybrid vigor. The seed care provided by seed producers and marketers was also important, including “(1) timely and careful selection in the fall, (2) careful storage over winter, (3) careful testing for germination and disease of seedlings, (4) grading of shelled seed to fit planter plates and, (5) dust treatment to help control disease in the planted seed” (Mosher, 1974, vol. 2, p. 220). What if he, Perry Holden, and other first-wave extension agents in the Corn Belt had been able to continue their work of educating farmers in the same caretaking practices that had contributed to the performance of commercially prepared hybrid seed?

Mosher did not think that open-pollinated varieties would have kept up with hybrid development. On the contrary, he believed that “they would have fallen far short in yield and quality of modern hybrids” (Mosher, 1974, vol. 2, p. 220). The regret palpable in *The Cornbelt's Last Open Pollinated Corn* is not for “lost” varieties but instead for the lost opportunity to see the corn extension work through to a different conclusion. Working again through his old data, Mosher was newly surprised at the extent to which his farmer-collaborators had been ignoring “the corn show winnings of their neighbors.” Corn shows, favoring visual qualities, were distinct from yield tests, which assessed production measures; by the 1910s they were mostly criticized by corn experts as leading to corn of lower quality (Fitzgerald, 1993, pp. 330–332). Assessing Woodford County farmers' corn 50 years on, Mosher newly saw that the farmers had been more influenced by the advice of those “making field and laboratory tests as guides” to developing productive and disease-resistant lines (Mosher, 1974, vol. 2, p. 212). In other words, demonstration and extension to improve seed care and therefore quality had been working. *The Cornbelt's Last Open Pollinated Corn* is itself a demonstration of this: an opportunity to see first-hand what farmers and extension agents had been able to accomplish in a little more than a decade with respect to understanding what made open-pollinated corn varieties perform well and what actions might increase corn production.

The decades following the Woodford County corn yield test saw Mosher continue to engage directly with farmers as an extension agent. The topic and nature of his advising shifted, however. He became a specialist in farm accounting and management, developing methods for helping farmers become better businessmen. His instructions were no longer about the quality and care of seeds but the importance of attentiveness to ledgers and account books. He optimistically judged this to have been at least as worthwhile as his corn work, with each of his various farm management projects adding “probably millions of dollars to income available for better family living” (Mosher, 1974, vol. 2, p. 225; on the history of farm management extension see Fitzgerald, 2003). However, those decades also saw him increasingly obsessed with the phenomenon of farm consolidation, as he watched bigger, more highly capitalized farmers buying out their smaller neighbors.^7^ Efforts to drive up the productivity of corn varieties had, if anything, exacerbated the problems of overproduction that plagued farmers in the 1920s and 1930s and privileged larger, wealthier producers. Similarly, making farmers into better businessmen may have contributed to efficiency and income generation on individual farms, but it likely also fostered the “industrial ideal” that drove many families out of farming altogether (Anderson, 2009; Conkin, 2008; Danbom, 2017; Fitzgerald, 2003).

As had been the case with Mosher's farmers' variety tests, which contributed to the gradual elimination of the very varieties they tested and celebrated, as well as the labor of making and maintaining them, the legacies of extension were complicated and in some sense contradictory. Mosher envisioned a Corn Belt that was, above all, populated, and only secondarily productive, characterized by “a rural life that would be socially attractive to young, middle aged and old, that would make satisfactory economic use of land, labor, capital and management, that would make full use of natural resources and also conserve them for future generations” (Mosher, 1974, vol. 2, p. 226). Without state and national regulations to support various aspects of that vision, his efforts to help individual farmers eke a little more living from the land ultimately served a different purpose. His values and those advanced through institutionalized extension activities were in deep and ultimately unresolvable tension.

Historians of agricultural extension in the United States have rightly characterized it as an enterprise that facilitated the industrialization, capitalization, and consolidation of farms. My account of corn demonstrations of the early 20th century is no exception. Observers then and now agree that this work was instrumental in the making of hybrid corn and the corn seed industry—and further agree that the US hybrid corn seed industry was critical to the privatization of crop development and seed production across crops and around the world by the 21st century. My account sustains claims that extension work has typically served the interests of larger growers rather than smaller ones, advancing solutions that worked at larger scales over those that addressed individual or even local needs. The corn yield tests were expanded and celebrated not because they identified techniques useful to individual farmers or varieties suited to a particular county, but because they surfaced strains of corn that were useful across counties and even states. They ultimately directed benefits to farmers with the resources to buy seeds of these strains and the ability to operate at scales that could overcome the ever-diminishing returns on producing too-abundant commodities. The resulting inequalities in experience and opportunity, challenging for many White settler farmers, would have had even greater ramifications for African American and Native American farmers, most of whom were not only economically marginalized but also subject to overt racial discrimination on the part of state and federal agricultural institutions.

Yet even as my account affirms these perspectives, revisiting this history through the ambitions and experiences of a single agricultural extension worker, in this case Martin Mosher, also offers the opportunity to see different objectives and possibilities for extension work moving forward. It presents an example of how the knowledge of farmers and labor of extension workers could converge in the short term to address local concerns, achieve local goals, and sustain local production. As researchers and institutions seek alternative agricultural models that address concerns about the social and ecological sustainability of industrial farming, they may do well to revisit the history of corn seed demonstrations and the ethos of local solutions they initially espoused. Perhaps there were, as Mosher tried in his final years to show, fruitful pathways forward from those programs left unexplored. However, in imagining such possibilities, today's researchers would also do well to observe how local objectives were ultimately undermined—and therefore recognize the broader institutional, political, and economic transformations necessary to realize these.

## Data Availability

Data sharing is not applicable to this article as no datasets were generated or analyzed during the current study.

## Footnotes

1 I first consulted the physical volumes at the special collections of the National Agricultural Library, Beltsville, MD, in 2017. The volumes have since been digitized and are now available via the Internet Archive. See the reference list for access information.

2 They also consistently privileged White settler farmers, with policies that consciously discriminated against African American farmers (Harris, 2008) and sought to eliminate Native American farming practices (Firkus, 2010). Although not the focus of my analysis, the role of extension in the racialization of “good” farming practices and the development of agriculture as a largely White enterprise in the United States is also pivotal to the history I recount in this article. Earlier histories of extension in the United States tended to take a rosier view of extension as a progressive and democratic program for enhancing the agricultural economy by delivering research-based knowledge to farmers. See, for example, Rasmussen (1989) and Rogers (1988).

3 Other accounts of extension similarly seek to show the complex dynamics of this work. See, for example, Conz (2019) on 20th-century Lesotho, Baur and Iles (2023) on 20th-century California, and Lindberg and Palmås (2013) on 21st century Sri Lanka.

4 Here and throughout, I draw on several sources of biographical information about Mosher: Finding Aid, Mosher Papers, https://findingaids.lib.iastate.edu/spcl/arch/rgrp/16-3-55.html; Martin Mosher, correspondence with Arthur Mosher, ca. 1935, Mosher Papers, https://n2t.net/ark:/87292/w98n70; Sullivan (2021); and Mosher (1962b, 1974).

5 Several accounts credit the growing popularity of demonstration in this period to a federally funded boll weevil control program launched in the southern states in 1903 and led by Seaman A. Knapp. See Scott (1970), Rasmussen (1989), and Mitchell and Lowe (1990).

6 Farmers' preferences for different corn varieties would also have varied according to the intended uses of their harvest. Growers who needed a reliable crop solely to feed their own livestock may not have prized yield as highly as those growing corn for cash sale.

7 Mosher's regret for the transformation of farm families and rural communities into agribusinesses is palpable in “Farmsteads of the United States,” a seven-volume compilation of photographs taken by Mosher and his wife between 1949 and 1963. In “Farmsteads,” the Moshers documented mid-century farm building architecture and the overall “landscaping and neatness of home sites” before these disappeared amid the great transformations in production he saw unfolding (Mosher, 1974, vol. 2, p. 226). A copy of this photographic collection is held in the Martin L. Mosher Manuscripts, US National Agricultural Library Special Collections (collection no. MS 109).
